# Aspartate Aminotransferase to Platelet Ratio Index and Fibrosis-4 Index for Detecting Liver Fibrosis in Patients With Autoimmune Hepatitis: A Meta-Analysis

**DOI:** 10.3389/fimmu.2022.892454

**Published:** 2022-05-18

**Authors:** Bingtian Dong, Yuping Chen, Guorong Lyu, Xiaocen Yang

**Affiliations:** ^1^ Department of Ultrasound, The Second Affiliated Hospital of Fujian Medical University, Quanzhou, China; ^2^ Department of Endocrinology, The Second Affiliated Hospital of Fujian Medical University, Quanzhou, China; ^3^ Department of Clinical Medicine, Quanzhou Medical College, Quanzhou, China; ^4^ Department of Ultrasound, Chenggong Hospital, Xiamen University, Xiamen, China

**Keywords:** autoimmune hepatitis, liver fibrosis, noninvasive methods, APRI, FIB-4

## Abstract

**Background:**

Aspartate aminotransferase-to-platelet ratio index (APRI) and fibrosis-4 index (FIB-4) are the two most widely studied noninvasive markers of liver fibrosis. We aimed to assess the diagnostic accuracy of APRI and FIB-4 for liver fibrosis in patients with autoimmune hepatitis (AIH) using liver biopsy as the reference standard.

**Methods:**

PubMed, EMBASE, Cochrane Library and Web of Science databases were searched for studies (published as of May 1st, 2021) that assessed the diagnostic performance of APRI and FIB-4 for liver fibrosis in AIH. The summary area under receiver operating characteristics curve (AUROC), sensitivity, specificity, diagnostic odds ratios were used to assess the diagnostic accuracy of APRI and FIB-4 for detecting liver fibrosis.

**Results:**

Fourteen studies (including 1015 patients) were selected with 13 studies each evaluating the use of APRI and FIB-4 for detecting different stages of fibrosis in AIH. For prediction of significant fibrosis, advanced fibrosis, and cirrhosis, the summary AUROC value was 0.66 [95% confidence interval (CI): 0.61–0.70], 0.71 (95% CI: 0.67–0.75), and 0.75 (95% CI: 0.71–0.79) for APRI, and the summary AUROC value was 0.75 (95% CI: 0.71–0.79), 0.73 (95% CI: 0.69–0.77) and 0.79 (95% CI: 0.75–0.82) for FIB-4, respectively. The summary sensitivity and specificity for diagnosis of significant fibrosis, advanced fibrosis, and cirrhosis were 90% and 36%, 78% and 55%, and 77% and 61% for APRI, and 70% and 70%, 65% and 70%, and 78% and 65% for FIB-4, respectively.

**Conclusions:**

APRI and FIB-4 showed suboptimal diagnostic performance for identifying liver fibrosis in AIH with mediocre sensitivity and specificity.

## Introduction

Autoimmune hepatitis (AIH) is a chronic inflammatory liver disease, which affects all ages, both genders, and all ethnicities (1, 2). The morbidity burden of AIH appears to be increasing across the world (3). In a recent systematic review by Lv et al. (4), the pooled global annual incidence and prevalence of AIH were 1.37 and 17.44 per 100,000 people, respectively. AIH could have a progressive course that gradually develops into cirrhosis, hepatocellular carcinoma, hepatic decompensation, and even death (5–7). In previous studies, approximately 7% of patients with AIH were cirrhotic at the time of diagnosis and these patients showed worse survival outcomes (8, 9). According to the European Association for the Study of the Liver (EASL) guidelines (8), diagnosis of liver fibrosis and cirrhosis is crucial to guide treatment strategies in patients with AIH.

Liver biopsy is considered the “gold standard” for the staging of liver fibrosis; however, it is an invasive procedure with some limitations, such as sampling error, intra- and interobserver variability, and risk of complications (10). Moreover, use of liver biopsy for dynamic evaluation of liver fibrosis stage is unfeasible in clinical practice (11, 12). These factors limit the use of liver biopsy for screening and regular follow-up (13). Therefore, development of alternative, noninvasive methods to stage liver fibrosis in these patients is a key imperative.

In clinical practice, there is a felt-need for a noninvasive method for diagnosis of liver fibrosis that is readily available, low-cost, reliable, and accurate; such a method can also be used for the follow-up monitoring of liver fibrosis (11, 14). Immunosuppressive treatment with corticosteroids alone or in combination with azathioprine is the mainstay of therapy for patients with AIH, which is associated with an excellent prognosis in most cases (15). The presence and extent of fibrosis are associated with the progression of the disease and response to treatment (13); thus, accurate assessment of the degree of liver fibrosis using noninvasive methods is also important for evaluating treatment response of AIH patients during the follow-up.

Currently, serum indices of liver fibrosis based on inexpensive laboratory tests have been developed, including the aspartate aminotransferase to platelet ratio index (APRI) and the fibrosis index based on the four factors (Fibrosis-4 index; FIB-4) (16, 17). In the last two decades, these two serum indices have been extensively studied and their diagnostic value for detecting liver fibrosis assessed in different populations (18–20). Of all the serum noninvasive tests for the evaluation of liver fibrosis, APRI and FIB-4 are the two most widely studied (11, 21).

Several recent meta-analyses have investigated the value of these two serum indices for detection of liver fibrosis. Lin et al. (22) conducted a meta-analysis of studies that investigated the use of APRI for diagnosing liver fibrosis caused by hepatitis C virus (HCV). Xiao et al. (11) conducted a meta-analysis to assess the comparative accuracy of APRI and FIB-4 for the diagnosis of hepatitis B virus-related liver fibrosis. In addition, Xiao et al. (23) recently conducted a meta-analysis to investigate the diagnostic accuracy of APRI and FIB-4 for staging liver fibrosis in patients with nonalcoholic fatty liver disease (NAFLD). Some recent studies that sought to externally validate these two noninvasive models in predicting liver fibrosis in patients with AIH have yielded inconsistent results. To the best of our knowledge, there is just one published meta-analysis that assessed the diagnostic performance of APRI and FIB-4 for the staging of fibrosis in AIH patients (24); however, this study only assessed the accuracy of these two serum indices in predicting advanced fibrosis and cirrhosis. In addition, only a limited number of studies were included in the previous meta-analysis.

Therefore, in the present study, we conducted a systemic review and meta-analysis with the aim to assess the diagnostic accuracy of APRI and FIB-4 for the assessment of liver fibrosis in patients with AIH, using liver biopsy as the reference standard. Moreover, we explored the sources of heterogeneity in results across studies using meta-regression analysis.

## Materials and Methods

### Search Strategy

The Preferred Reporting Items for Systematic Reviews and Meta-Analyses (PRISMA) guidelines were followed for reporting this systemic review and meta-analysis (25). An online literature search was conducted on PubMed, EMBASE, the Cochrane Library, and the Web of Science databases for articles published as of May 1st, 2021 using the following keywords: APRI, AST-to-platelet ratio index, AST, platelet, FIB-4, autoimmune hepatitis, liver fibrosis and cirrhosis. In addition, reference lists of the included articles were manually screened to identify other relevant publications.

### Study Selection

Studies were included according to the following criteria: (1) studies that assessed the diagnostic value of APRI and/or FIB-4 for the assessment of liver fibrosis in patients with AIH; (2) use of liver biopsy as the reference standard; (3) availability of adequate data to construct at least one 2 × 2 contingency table; and (4) sample size larger than 10 AIH patients, given its prevalence. The exclusion criteria were: (1) review articles, conference abstracts, comments, editorials, case reports, and letters; (2) studies unrelated to the topic; (3) animal or basic research; (4) duplicate publications; and (5) studies not published in English language.

### Data Extraction and Quality Assessment

Two investigators (DBT and CYP) independently conducted the literature search, selected the articles for inclusion, and extracted the data. A standardized data extraction format was prepared for this study using Microsoft Excel 2019. Data pertaining to the following variables were extracted from the included studies: number of patients, age, sex, liver biopsy scoring system, number of patients with different stages of liver fibrosis as well as cutoff values, sensitivity, specificity, positive predictive value, negative predictive value and area under the receiver operating characteristic curve (AUROC) values for detecting significant fibrosis, advanced fibrosis, and cirrhosis. In the present study, significant fibrosis, advanced fibrosis, and cirrhosis were defined as stages F2-F4, F3-F4, and F4, respectively, according to the METAVIR or Scheuer scoring system.

The revised Quality Assessment of Diagnostic Accuracy Studies-2 (QUADAS-2) tool was used to assess the quality of the studies included in this meta-analysis (26). Study eligibility and quality were independently evaluated by the two researchers (DBT and CYP); discrepancies, if any, were resolved by consensus or by participation of a third investigator (LGR).

### Data Synthesis and Analysis

The objective of this meta-analysis was to assess the diagnostic accuracy of APRI and FIB-4 for the assessment of liver fibrosis in patients with AIH. Data extracted from the included studies were used to calculate the summary sensitivity and specificity, positive likelihood ratio (LR), negative LR, diagnostic odds ratios (DORs) and the corresponding 95% confidence intervals (CIs). Using the data, the summary receiver operating characteristic curves of these two serum indices were also constructed, and then the summary AUROC values were obtained. Moreover, the summary sensitivity and specificity and the summary DORs were calculated to further evaluate the accuracy of these two serum indices for predicting liver fibrosis in AIH patients. A random-effect coefficient binary regression model was used to calculate the summary sensitivity and specificity.

All statistical analyses were performed using Stata 15.0 (STATA, College Station, Texas, USA) and Meta-Disc Version 1.4 (Hospital Ramony Cajal, Madrid, Spain).

### Assessment of Heterogeneity and Publication Bias

Heterogeneity among the included studies with respect to the diagnostic accuracy of APRI and FIB-4 was assessed using the Cochrane-Q test, and the inconsistency index *I* ^2^ was also calculated. *I* ^2^ value > 50% is suggestive of substantial heterogeneity. Moreover, meta-regression and subgroup analyses were conducted to explore the potential sources of heterogeneity using the following covariates: (1) year of publication; (2) region; (3) sample size; (4) median/mean age; and (5) percentage of advanced fibrosis.

To assess the influence of potential publication bias on the results of the meta-analysis, a linear regression test of funnel plot asymmetry using Deeks’ plot was conducted.

## Results

### Search Results

**Figure 1:**
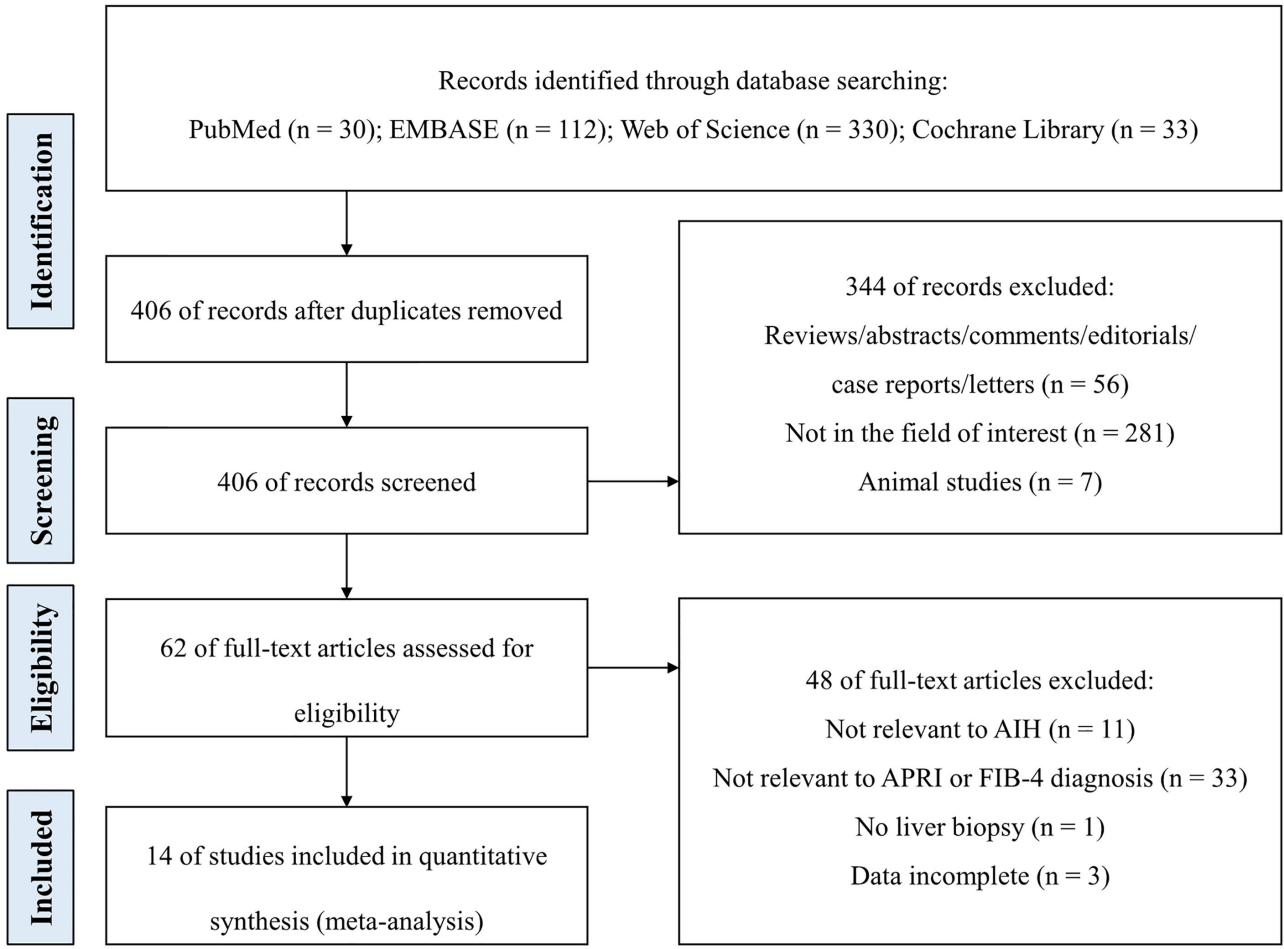
The study flow diagram.

**Figure 1** illustrates the study selection process. A total of 505 studies were retrieved on database search using the search strategy. After removal of 99 duplicate publications, titles and abstracts of 406 studies were screened. Of these, 392 studies did not qualify the inclusion criteria. Finally, 14 studies were included in the meta-analysis after full-text review (13, 27–39).

### Characteristics of the Included Studies

The characteristics of the included studies are summarized in **Table 1**. In total, thirteen APRI original articles with 2106 AIH patients and thirteen FIB-4 original articles with 2112 AIH patients were selected for evaluation and meta-analysis. The mean age of patients was 51.7 years (range: 40.0–64.0). In 9 (64.3%) studies, the average age of patients with AIH was above fifty years. Male patients accounted for approximately 20.2% (range: 11.1%–41.5%) of all patients. It is worth mentioning that the average proportion of males in the individual studies was much lower than that of females. A total of 7 (50.0%) studies were published between 2018 and 2020, 6 (42.9%) studies were published between 2016 and 2017, and 1 (7.1%) study was published in 2006. Most of the included studies were conducted in Asia (8 in China, 1 in Saudi Arabia, 1 in Japan, and 1 in Korea); 2 studies were conducted in Germany and 1 study was conducted in the USA. With the exception of 1 (7.1%) study that included subjects from three centers, all other studies were single-center studies. Among the included studies, 4 (28.6%) studies were prospective, and 10 (71.4%) studies were retrospective. Eleven studies used the METAVIR score, and 2 of the remaining 3 studies used the Scheuer score to determine the stages of liver fibrosis in AIH. The articles included in the meta-analysis were published in twelve different Science Citation Index journals; the mean impact factor of these journals was 3.160 (range: 0.660–5.742).

**Table 1:** Main characteristics of studies included in this meta-analysis.

Author, Year, Region	Models	Range time of study	Diagnostic criteria	Center	Study design	n	Interval between biopsy and blood test	Median/mean age, years (male %)	Liver biopsy scoring system	Blind	Liver biopsy length (mm)	Significant fibrosis, advanced fibrosis, cirrhosis (%)
Abdo, 2006, Saudi Arabia (27)	APRI	1996−2004	IAIHG 1999	One	Retrospective	39	NA	45.4 (35.0)	METAVIR	NA	NA	NA, 38.5, NA
Anastasiou et al., 2016, Germany (28)	APRI, FIB-4	2008−2013	IAIHG 2008	One	Retrospective	53	NA	47.3 (41.5)	METAVIR	Yes	≥ 14	83.0, 54.7, 28.3
Nishikawa et al., 2016, Japan (29)	APRI, FIB-4	2005−2015	IAIHG 1999	One	Prospective	84	NA	64.0 (17.9)	METAVIR	NA	NA	78.6, 50.0, 21.4
Sheptulina et al., 2016, Germany (30)	APRI, FIB-4	2008−2014	IAIHG 1999	One	Prospective	76	7 days	40.0 (14.5)	METAVIR	Yes	≥ 14	72.4, 48.7, 38.2
Guo et al., 2017, China (31)	APRI, FIB-4	2012−2017	IAIHG 2008	One	Retrospective	108	Same day	46.5 (18.5)	METAVIR	NA	≥ 15	72.2, 50.0, 22.2
Wang et al., 2017, USA (32)	APRI, FIB-4	2007−2015	IAIHG 1999	One	Retrospective	36	NA	51.6 (NA)	METAVIR	Yes	NA	72.2, 52.8, 36.1
Xu et al., 2017, China (33)	APRI, FIB-4	2014−2016	IAIHG 2008	One	Prospective	100	NA	45.0 (19.0)	METAVIR	Yes	≥ 10	84.0, 50.0, 23.0
Zeng et al., 2018, China (34)	APRI, FIB-4	2012−2017	AASLD 2010	One	Prospective	76	NA	56.8 (21.1)	NA	Yes	NA	NA, NA, 50.0
Liu et al., 2019, China (35)	FIB-4	2008−2018	IAIHG 1999	One	Retrospective	45	Same day	54.3 (15.6)	METAVIR	Yes	≥ 10	NA, 48.9, NA
Park et al., 2019, Korea (36)	APRI, FIB-4	2014-2017	IAIHG 1999	One	Retrospective	49	NA	56.0 (14.3)	METAVIR	Yes	NA	63.3, 42.9, 22.4
Yuan et al., 2019, China (37)	APRI, FIB-4	2010−2017	IAIHG 1999	One	Retrospective	55	7 days	56.7 (16.4)	METAVIR	Yes	≥ 10	76.4, 49.1, 18.2
Li et al., 2020, China (38)	APRI, FIB-4	2010−2019	IAIHG 1999	One	Retrospective	72	NA	54.0 (11.1)	METAVIR	Yes	NA	NA, 37.5, NA
Wang et al., 2020, China (39)	APRI, FIB-4	2016−2019	AASLD 2010	Three	Retrospective	119	7 days	52.5 (16.8)	Scheuer	Yes	NA	68.9, 28.6, 10.1
Xing et al., 2020, China (13)	APRI, FIB-4	2016−2019	IAIHG 1999	One	Retrospective	103	NA	54.0 (21.4)	Scheuer	Yes	≥ 15	84.5, 39.8, 30.1

AASLD, American Association for the Study of Liver Diseases; APRI, aspartate aminotransferase to platelet ratio index; FIB-4, fibrosis index based on the four factors; IAIHG, International Autoimmune Hepatitis Group; NA, not available.

**Figure 2:**
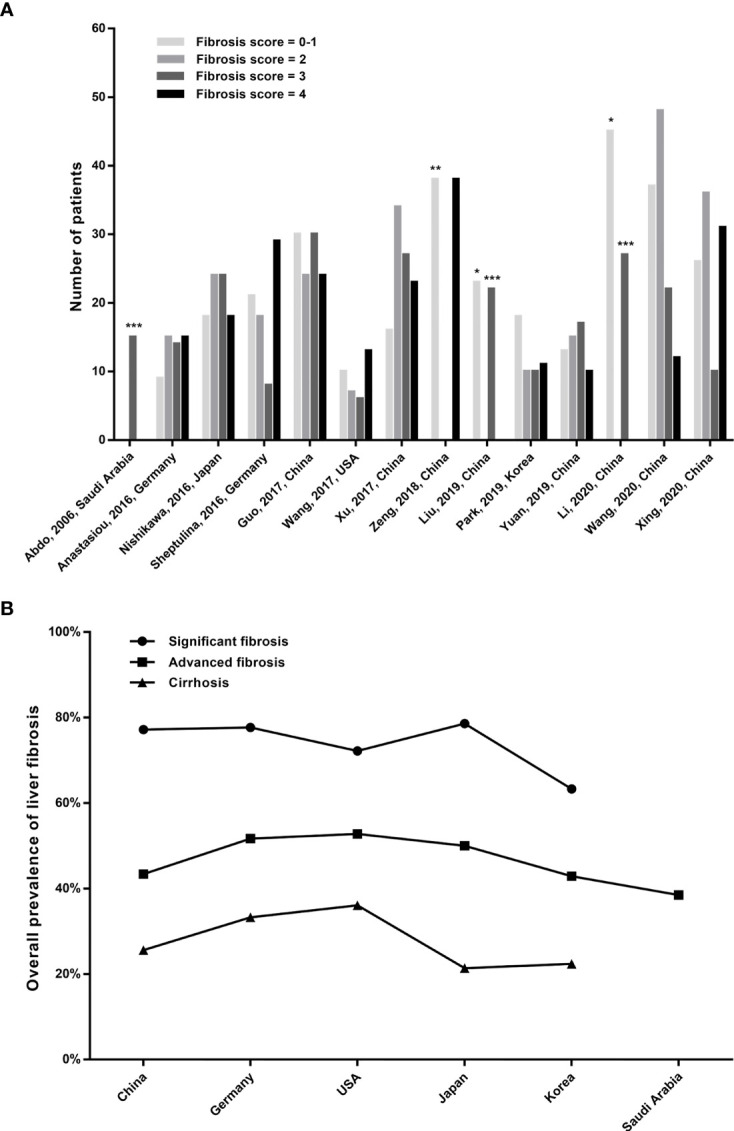
**(A)** Distribution of liver fibrosis stages in AIH patients of the included studies. *The columns represent fibrosis stage of 0-2; **the column represents fibrosis stage of 0-3; and ***the columns represent fibrosis stage of 3-4. **(B)** The overall prevalence of fibrosis stages between the included regions.

The prevalence of liver fibrosis in patients with AIH across studies is shown in **Table 1**, and **Figure 2** displays the distribution of liver fibrosis stages in each included study as well as the region-wise overall prevalence of fibrosis stages. The total prevalence of significant fibrosis, advanced fibrosis, and cirrhosis was 75.6% (range: 63.3%–84.5%), 45.5% (range: 28.6%–54.7%), and 27.3% (range: 10.1%–50.0%).

### Diagnostic Accuracy for Significant Fibrosis

A total of six studies (508 patients) investigated the diagnostic performance of APRI and FIB-4 for the detection of significant fibrosis (**Table 2**). The mean AUROC values of APRI and FIB-4 for diagnosing significant fibrosis were 0.580 (range: 0.499–0.635) and 0.662 (range: 0.560–0.750), respectively. Results were then combined, the summary AUROC value of FIB-4 for diagnosing significant fibrosis was 0.75 (95% CI: 0.71–0.79), while the summary AUROC value of APRI was 0.66 (95% CI: 0.61–0.70) (**Table 3**). APRI showed a good summary sensitivity (90%), but had a poor summary specificity (36%). The summary sensitivity and specificity of FIB-4 for predicting significant fibrosis in AIH patients were both 70%. Moreover, the summary DORs of APRI and FIB-4 for diagnosing significant fibrosis were 5 (95% CI: 2–10) and 5 (95% CI: 3–8), respectively. Notably, the cutoff values varied among the included studies. A total of five studies had simultaneously reported the cutoff values of APRI and FIB-4 for the diagnosis of significant fibrosis. The average cutoff values of APRI and FIB-4 for predicting significant fibrosis were 0.89 (range: 0.27–1.55) and 2.90 (range: 1.28–5.07), respectively.

**Table 2:** APRI and FIB-4 diagnostic performance for detecting liver fibrosis in AIH.

Study	Model	Diagnostic criterion (cutoff point)	Sensitivity, %	Specificity, %	AUROC
Abdo, 2006, Saudi Arabia* (27)	APRI	AF: 1.5	AF: 66	AF: 67	AF: 0.690
Anastasiou et al., 2016, Germany (28)	APRI FIB-4	SF: 1.45, AF: 1.24, Cirrhosis: 1.848 SF: 3.2, AF: 1.93, Cirrhosis: 2.61	SF: 59.1, AF: 69.0, Cirrhosis: 81.8 SF: 47.7, AF: 75.9, Cirrhosis: 90.9	SF: 67, AF: 50, Cirrhosis: 57.1 SF: 88.9: AF: 50, Cirrhosis: 59.5	SF: 0.601, AF: 0.527, Cirrhosis: 0.665 SF: 0.659, AF: 0.614, Cirrhosis: 0.766
Nishikawa et al., 2016, Japan (29)	APRI FIB-4	AF: 0.9, Cirrhosis: 2.0 AF: 5.1, Cirrhosis: 3.4	AF: 83.3, Cirrhosis: 61.1 AF: 52.4, Cirrhosis: 100	AF: 52.4, Cirrhosis: 78.8 AF: 90.4, Cirrhosis: 63.7	AF: 0.698, Cirrhosis: 0.744 AF: 0.747, Cirrhosis: 0.843
Sheptulina et al., 2016, Germany (30)	APRI FIB-4	AF: 0.84, Cirrhosis: 1.95 AF: 2.37, Cirrhosis: 2.59	SF: 93.9, AF: 82.9, Cirrhosis: 70.4 SF: 72.3, AF: 74.3, Cirrhosis: 77.8	SF: 31.9, AF: 62.5, Cirrhosis: 77.5 SF: 74.0, AF: 70.6, Cirrhosis: 71.4	SF: 0.626, AF: 0.707, Cirrhosis: 0.723 SF: 0.702, AF: 0.742, Cirrhosis: 0.795
Guo et al., 2017, China (31)	APRI FIB-4	SF: 0.88, AF: 2.13, Cirrhosis: 1.50 SF: 2.90, AF: 3.21, Cirrhosis: 2.72	SF: 75.6, AF: 42.6, Cirrhosis: 70.8 SF: 51.3, AF: 23.7, Cirrhosis: 66.7	SF: 53.3, AF: 88.9, Cirrhosis: 64.3 SF: 83.3, AF: 77.8, Cirrhosis: 63.1	SF: 0.635, AF: 0.645, Cirrhosis: 0.713 SF: 0.659, AF: 0.636, Cirrhosis: 0.658
Wang et al., 2017, USA (32)	APRI FIB-4	NA NA	AF: 99.8, Cirrhosis: 89.6 AF: 69.8, Cirrhosis: 68.9	AF: 54.8, Cirrhosis: 62.8 AF: 75.8, Cirrhosis: 74.5	AF: 0.728, Cirrhosis: 0.776 AF: 0.786, Cirrhosis: 0.803
Xu et al., 2017, China (33)	APRI FIB-4	NA NA	AF: 79.5 AF: 71.3	AF: 48.0 AF: 77.9	AF: 0.637 AF: 0.793
Zeng et al., 2018, China (34)	APRI FIB-4	NA NA	Cirrhosis: 81.5 Cirrhosis: 68.4	Cirrhosis: 55.3 Cirrhosis: 86.8	Cirrhosis: 0.666 Cirrhosis: 0.825
Liu et al., 2019, China (35)	FIB-4	AF: 2.26	AF: 77.3	AF: 73.9	AF: 0.757
Park et al., 2019, Korea (36)	APRI FIB-4	SF: 0.32, AF: 0.91, Cirrhosis: 3.58 SF: 1.28, AF: 1.64, Cirrhosis: 2.68	SF: 96.8, AF: 85.7, Cirrhosis: 100 SF: 96.8, AF: 90.5, Cirrhosis: 72.7	SF: 27.8, AF: 46.4, Cirrhosis: 44.7 SF: 33.3, AF: 35.7, Cirrhosis: 42.1	SF: 0.55, AF: 0.59, Cirrhosis: 0.38 SF: 0.56, AF: 0.66, Cirrhosis: 0.55
Yuan et al., 2019, China (37)	APRI FIB-4	NA NA	Cirrhosis: 79.8 Cirrhosis: 89.8	Cirrhosis: 74.5 Cirrhosis: 74.7	Cirrhosis: 0.798 Cirrhosis: 0.881
Li et al., 2020, China (38)	APRI FIB-4	AF: 1.896 AF: 5.104	AF: 74.1 AF: 63.0	AF: 48.9 AF: 73.3	AF: 0.579 AF: 0.702
Wang et al., 2020, China (39)	APRI FIB-4	SF: 0.271, AF: 0.381, Cirrhosis: 0.547 SF: 2.055, AF: 3.928, Cirrhosis: 2.212	SF: 98.8, AF: 94.1, Cirrhosis: 83.3 SF: 70.7, AF: 44.1, Cirrhosis: 75.0	SF: 11.1, AF: 19.1, Cirrhosis: 24.5 SF: 58.3, AF: 66.7, Cirrhosis: 42.5	SF: 0.499, AF: 0.434, Cirrhosis: 0.414 SF: 0.639, AF: 0.522, Cirrhosis: 0.535
Xing et al., 2020, China (13)	APRI FIB-4	SF: 1.55, AF: 2.18, Cirrhosis: 1.81 SF: 5.07, AF: 5.6, Cirrhosis: 6.44	SF: 81.8, AF: 48.8, Cirrhosis: 45.2 SF: 67.5, AF: 65.9, Cirrhosis: 67.7	SF: 42.3, AF: 69.4, Cirrhosis: 75.0 SF: 73.1, AF: 61.3, Cirrhosis: 63.9	SF: 0.57, AF: 0.57, Cirrhosis: 0.56 SF: 0.75, AF: 0.63, Cirrhosis: 0.66

AF, advanced fibrosis; AIH, autoimmune hepatitis; APRI, aspartate aminotransferase to platelet ratio index; AUROC, area under the receiver operating characteristic curve; FIB-4, fibrosis index based on the four factors; SF, significant fibrosis.

*The data was selected at a cutoff value of 1.5.

NA, not available.

**Table 3:** Meta-analysis results of APRI and FIB-4 for prediction of significant fibrosis, advanced fibrosis and cirrhosis in AIH.

	Number of Studies (Patients)	Cutoff Value(Mean, Range)	Summary Sensitivity (95% CI, %)	Summary Specificity (95% CI, %)	Summary LR+ (95% CI)	Summary LR-(95% CI)	Summary AUROC (95% CI)	Summary DOR (95% CI)
**Significant fibrosis**								
APRI	6 (508)	0.89 (0.27−1.55)	90 (74−97)	36 (21−55)	1.4 (1.2−1.7)	0.28 (0.14−0.57)	0.66 (0.61−0.70)	5 (2−10)
FIB-4	6 (508)	2.90 (1.28−5.07)	70 (54−82)	70 (52−83)	2.3 (1.6−3.3)	0.43 (0.31−0.60)	0.75 (0.71−0.79)	5 (3−8)
**Advanced fibrosis**								
APRI	11 (839)	1.33 (0.38−2.18)	78 (66−86)	55 (42−66)	1.7 (1.4−2.0)	0.41 (0.30−0.55)	0.71 (0.67−0.75)	4 (3−6)
FIB-4	11 (845)	3.46 (1.64−5.60)	65 (53−76)	70 (61−77)	2.1 (1.7−2.7)	0.50 (0.38−0.67)	0.73 (0.69−0.77)	4 (3−7)
**Cirrhosis**								
APRI	10 (759)	1.89 (0.55−3.58)	77 (65−86)	61 (50−72)	2.0 (1.6−2.5)	0.38 (0.27−0.54)	0.75 (0.71−0.79)	5 (3−8)
FIB-4	10 (759)	3.24 (2.21−6.44)	78 (69−84)	65 (56−73)	2.2 (1.7−2.8)	0.35 (0.24−0.50)	0.79 (0.75−0.82)	6 (4−11)

AIH, autoimmune hepatitis; APRI, aspartate aminotransferase to platelet ratio index; AUROC, area under the receiver operating characteristic curve; CI, confidence interval; DOR, diagnostic odds ratio; FIB-4, fibrosis index based on the four factors; LR+, positive likelihood ratio, LR-, negative likelihood ratio.

### Diagnostic Accuracy for Advanced Fibrosis

**Figure 3:**
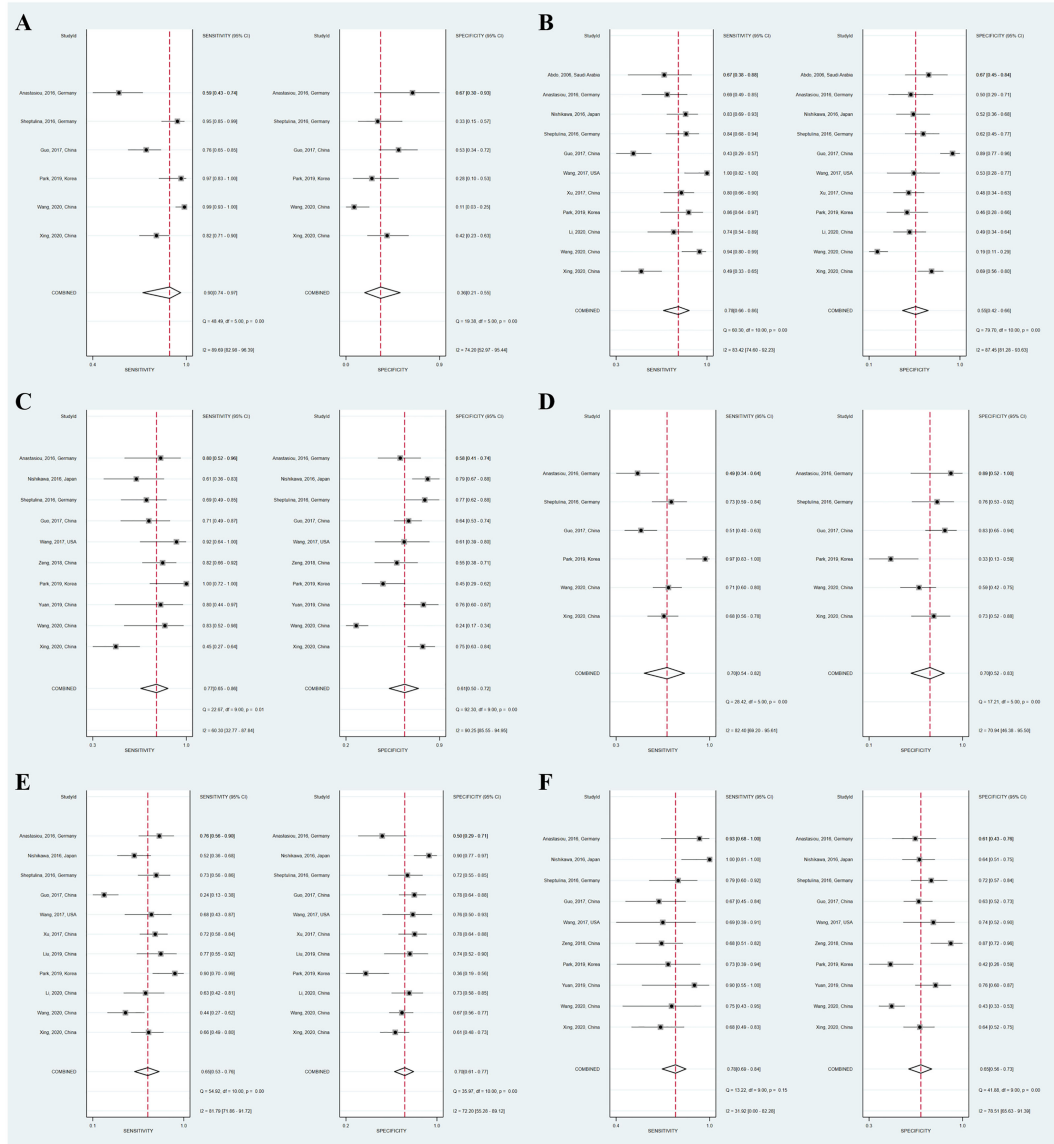
Coupled forest plots of the summary sensitivity and specificity of APRI and FIB-4 for the prediction of liver fibrosis in AIH patients. **(A)** APRI for detecting significant fibrosis; **(B)** APRI for detecting advanced fibrosis; **(C)** APRI for detecting cirrhosis; **(D)** FIB-4 for detecting significant fibrosis; **(E)** FIB-4 for detecting advanced fibrosis; **(F)** FIB-4 for detecting cirrhosis.

**Figure 4:**
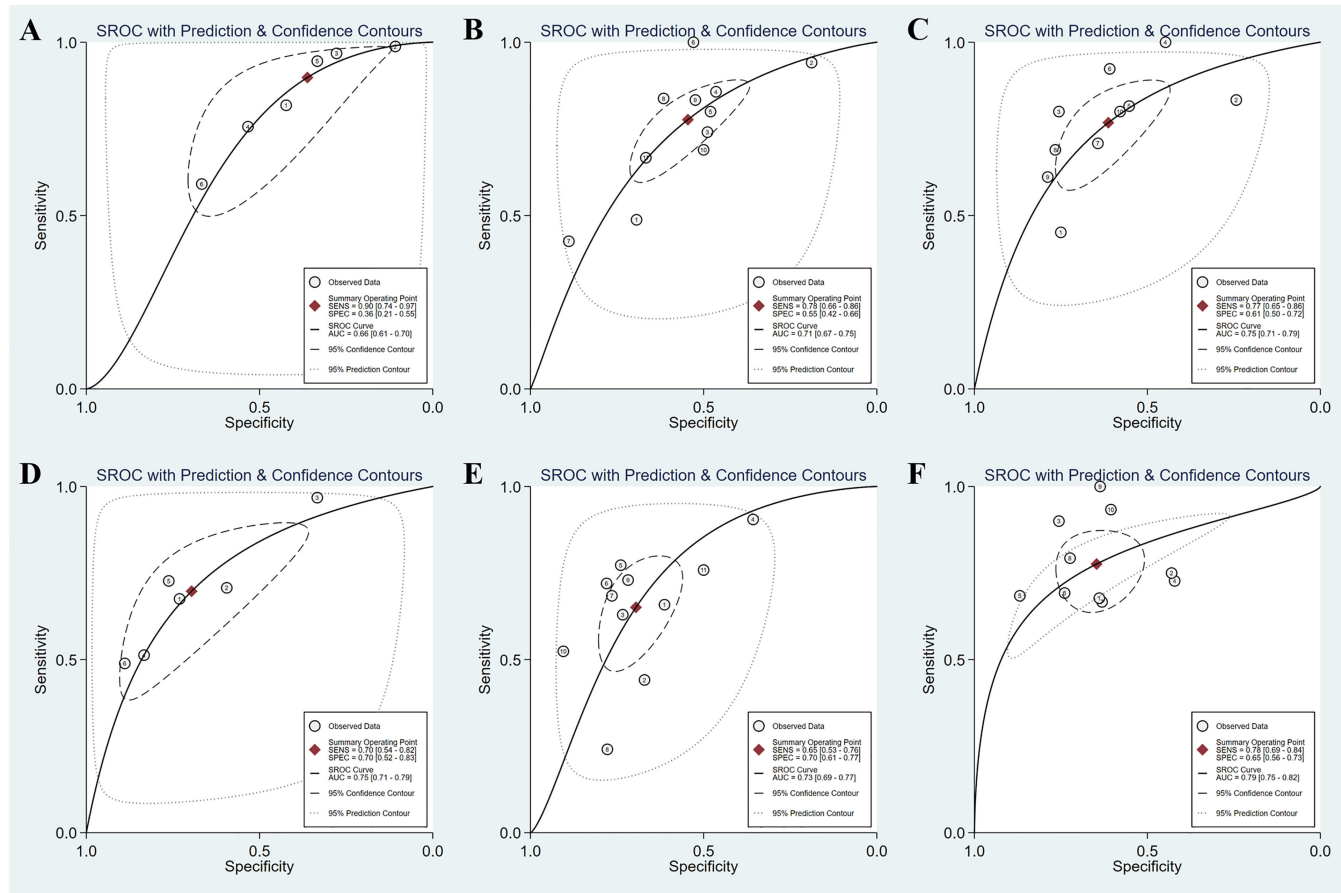
SROC curves of the diagnostic performance of APRI and FIB-4 for the prediction of liver fibrosis in AIH patients. **(A)** APRI for detecting significant fibrosis; **(B)** APRI for detecting advanced fibrosis; **(C)** APRI for detecting cirrhosis; **(D)** FIB-4 for detecting significant fibrosis; **(E)** FIB-4 for detecting advanced fibrosis; **(F)** FIB-4 for detecting cirrhosis.

Twelve studies (with 884 AIH patients) examined the ability of these two serum indices in predicting advanced fibrosis. Among these studies, 11 (839 patients) and 11 (845 patients) studies investigated the diagnostic performance of APRI and FIB-4 for advanced fibrosis. The summary sensitivity of APRI for detecting advanced fibrosis exceeded 75%; however, the summary specificity of APRI was mediocre (**Figure 3**). The summary sensitivity and specificity of FIB-4 for detecting advanced fibrosis were 65% and 70%, respectively. The summary specificity of FIB-4 in predicting advanced fibrosis was greater than that of APRI (70% vs. 55%), but the summary sensitivity was lower than that of APRI (65% vs. 78%). The summary AUROC values of APRI and FIB-4 for diagnosing advanced fibrosis were 0.71 (95% CI: 0.67–0.75) and 0.73 (95% CI: 0.69–0.77), respectively (**Figure 4**). The mean AUROC values of APRI and FIB-4 for diagnosing advanced fibrosis were 0.619 (range: 0.434–0.728) and 0.690 (range: 0.522–0.793), respectively. The summary DORs of APRI and FIB-4 for predicting advanced fibrosis were 4 (95% CI: 3–6) and 4 (95% CI: 3–7), respectively (**Table 3**).

### Diagnostic Accuracy for Cirrhosis

Ten (759 patients) studies had also examined the diagnostic performance of APRI and FIB-4 for predicting cirrhosis in AIH. The summary sensitivities of APRI and FIB-4 for detecting cirrhosis were 77% (95% CI: 65%–86%) and 78% (95% CI: 69%–84%), respectively (**Table 3**). The summary specificities of APRI and FIB-4 for detecting cirrhosis were 61% (95% CI: 50%–72%) and 65% (95% CI: 56%–73%), respectively. Moreover, the summary AUROC values of APRI and FIB-4 for the diagnosis of cirrhosis were 0.75 (95% CI: 0.71–0.79) and 0.79 (95% CI: 0.75–0.82), respectively. We found that both APRI and FIB-4 had greater summary AUROC values in detecting cirrhosis than detecting significant fibrosis and advanced fibrosis. The mean AUROC values of APRI and FIB-4 for diagnosing cirrhosis were 0.644 (range: 0.380–0.798) and 0.732 (range: 0.535−0.881), respectively (**Table 2**). In addition, the summary DORs of APRI and FIB-4 were 5 (95% CI: 3–8) and 6 (95% CI: 4–11), respectively. The summary positive LR values of APRI and FIB-4 for the diagnosis of cirrhosis were 2.0 (95% CI: 1.6–2.5) and 2.2 (95% CI: 1.7–2.8), respectively.

### Heterogeneity and Publication Bias

We observed substantial heterogeneity in several groups (**Figure 3**). Substantial heterogeneity was observed with respect to the summary sensitivity (*I* ^2^ = 89.69%, 83.42%, and 60.30%) and summary specificity (*I* ^2^ = 74.20%, 87.45%, and 90.25%) of APRI for detecting significant fibrosis, advanced fibrosis, and cirrhosis, respectively. Similarly, substantial heterogeneity was observed with respect to the summary sensitivity (*I* ^2^ = 82.40% and 81.79%) and specificity (*I* ^2^ = 70.94% and 72.20%) of FIB-4 for detecting significant fibrosis and advanced fibrosis, respectively. Substantial heterogeneity was also observed with regard to the summary specificity (*I* ^2^ = 78.51%), but not with regard to the summary sensitivity, when FIB-4 was used to detect cirrhosis. The conduct of meta-regression analyses is limited by the number of studies. In groups of larger than ten studies, methodological heterogeneity can be examined. The accuracy of APRI for the diagnosis of advanced fibrosis was not affected by the median/mean age [≥ 51.6 (median of all included studies) vs. < 51.6] (*P* = 0.83), sample size [≥ 76 (median of all included studies) vs. < 76] (*P* = 0.49), year of publication [≥ 2017 (median of all included studies) vs. < 2017] (*P* = 0.30), region (China vs. Non-China) (*P* = 0.50), or percentage of advanced fibrosis [≥ 48.7% (median of all included studies) vs. < 48.7%] (*P* = 0.18). Moreover, the diagnostic accuracy of FIB-4 for advanced fibrosis was not affected by the following factors: sample size [≥ 76 (median of all included studies) vs. < 76] (*P* = 0.91), year of publication [≥ 2017 (median of all included studies) vs. < 2017] (*P* = 0.83), median/mean age [≥ 52.5 (median of all included studies] vs. < 52.5] (*P* = 0.50), percentage of advanced fibrosis [≥ 48.9% (median of all included studies) vs. < 48.9%] (*P* = 0.49), or region (China vs. Non-China) (*P* = 0.35). Results of subgroup analyses of APRI and FIB-4 for the diagnosis of advanced fibrosis are shown in **Tables 4**, **5**.

**Table 4:** Subgroup analysis of APRI in prediction of advanced fibrosis.

Parameter	Subgroup	Number of Studies (Patients)	Summary Sensitivity (95% CI, %)	Summary Specificity (95% CI, %)	Summary AUROC (95% CI)
Year of publication	≥ 2017^a^	7 (587)	78 (60-89)	52 (34-70)	0.71 (0.66-0.74)
	< 2017	4 (252)	78 (70-84)	57 (49-66)	0.74 (0.70-0.78)
Region	China	5 (502)	72 (50-87)	56 (32-77)	0.69 (0.65-0.73)
	Non-China	6 (337)	82 (73-89)	55 (48-62)	0.60 (0.56-0.65)
Sample size	≥ 76^a^	6 (590)	76 (57-89)	57 (36-75)	0.72 (0.68-0.76)
	< 76	5 (249)	80 (66-90)	52 (43-60)	0.56 (0.52-0.61)
Median/mean age	≥ 51.6^a^	6 (463)	84 (67-93)	45 (32-60)	0.67 (0.63-0.71)
	< 51.6	5 (376)	70 (55-81)	66 (49-79)	0.73 (0.69-0.77)
Percentage of advanced fibrosis	≥ 48.7%^a^	6 (457)	78 (63-88)	60 (45-74)	0.75 (0.71-0.78)
	< 48.7%	5 (382)	77 (56-90)	48 (31-66)	0.66 (0.62-0.70)

APRI, aspartate aminotransferase to platelet ratio index; AUROC, area under the receiver operating characteristic curve; CI, confidence interval.

a Median of included APRI studies predicting advanced fibrosis.

**Table 5:** Subgroup analysis of FIB-4 in prediction of advanced fibrosis.

Parameter	Subgroup	Number of Studies (Patients)	Summary Sensitivity (95% CI, %)	Summary Specificity(95% CI, %)	Summary AUROC (95% CI)
Year of publication	≥ 2017^a^	8 (632)	64 (48-78)	68 (59-75)	0.71 (0.67-0.75)
	< 2017	3 (213)	66 (56-75)	74 (65-82)	0.762
Region	China	6 (547)	58 (41-72)	71 (65-76)	0.71 (0.67-0.75)
	Non-China	5 (298)	73 (59-84)	68 (46-84)	0.77 (0.73-0.80)
Sample size	≥ 76^a^	6 (590)	55 (40-70)	74 (66-81)	0.73 (0.69-0.77)
	< 76	5 (255)	75 (64-84)	62 (47-76)	0.76 (0.72-0.79)
Median/mean age	≥ 52.5^a^	6 (472)	66 (52-78)	68 (53-80)	0.72 (0.68-0.75)
	< 52.5	5 (373)	63 (43-79)	72 (64-79)	0.74 (0.70-0.78)
Percentage of advanced fibrosis	≥ 48.9%^a^	6 (426)	62 (44-77)	76 (65-84)	0.77 (0.73-0.80)
	< 48.9%	5 (419)	69 (52-82)	62 (51-72)	0.69 (0.64-0.73)

AUROC, area under the receiver operating characteristic curve; CI, confidence interval; FIB-4, fibrosis index based on the four factors.

a Median of included FIB-4 studies predicting advanced fibrosis.

**Figure 5:**
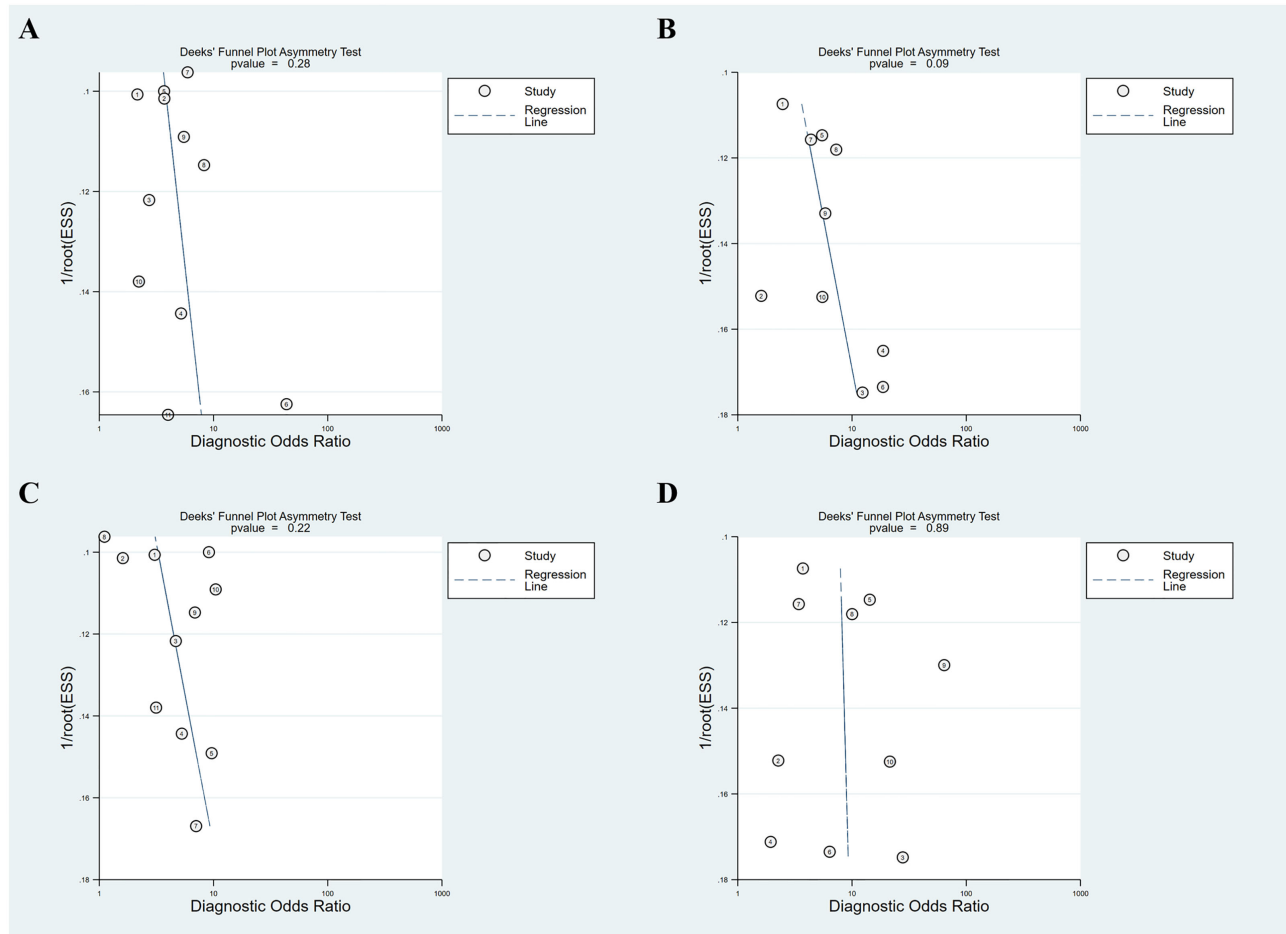
Deeks’ funnel plot asymmetry test for publication bias. **(A)** APRI for detecting advanced fibrosis; **(B)** APRI for detecting cirrhosis; **(C)** FIB-4 for detecting advanced fibrosis; **(D)** FIB-4 for detecting cirrhosis.

**Figure 5** illustrates the Deeks’ funnel plots of these two serum indices. We found no significant effect of publication bias on the meta-analysis of the diagnostic value of APRI for detecting advanced fibrosis and cirrhosis (*P* = 0.28 and 0.09). Similarly, no significant effect of publication bias was observed on the meta-analysis of the value of FIB-4 in detecting advanced fibrosis and cirrhosis (*P* = 0.22 and 0.89). Owing to the small number of included studies (i.e., < 10 studies), we did not further assess the effect of potential publication bias on the diagnostic value of APRI and FIB-4 for detecting significant fibrosis.

## Discussion

In this study, we performed a meta-analysis of available data pertaining to the diagnostic accuracy of APRI and FIB-4 for detecting different stages of liver fibrosis in AIH patients. Finally, a total of 13 APRI original articles with 2106 AIH patients and 13 FIB-4 original articles with 2112 AIH patients were systematic reviewed. Our results suggest a suboptimal diagnostic performance of APRI and FIB-4 for identifying significant fibrosis, advanced fibrosis, and cirrhosis in patients with AIH, with mediocre sensitivity and specificity.

Accurate diagnosis of liver fibrosis stage, particularly by noninvasive methods, plays a critical role in the assessment of disease progression in patients with AIH (24). Until now, there is no clear consensus about the diagnostic value of APRI and FIB-4 in predicting liver fibrosis in patients with AIH. A systematic review by Wu et al. (24) investigated the diagnostic performance of several noninvasive methods (including imaging techniques and serum indices) for evaluating liver fibrosis in patients with AIH. The reported summary AUROC of APRI and FIB-4 were 0.74 and 0.76 for the diagnosis of advanced fibrosis; 0.75 and 0.66 for the diagnosis of cirrhosis, respectively. The summary AUROC values of APRI and FIB-4 for the diagnosis of advanced fibrosis in that study were similar to our results; however, the summary AUROC value of FIB-4 for the diagnosis of cirrhosis in the previous study was significantly lower. This may be related to the inclusion of fewer studies with regard to the APRI (*n* = 8) and FIB-4 (*n* = 6) in the study by Wu et al. Moreover, Wu et al. did not report the summary AUROC values of APRI and FIB-4 for diagnosing significant fibrosis. In contrast, a total of 13 APRI original articles and 13 FIB-4 original articles were included in our study. Therefore, our study provides a more comprehensive analysis of the summary diagnostic accuracy of APRI and FIB-4 for detecting significant fibrosis, advanced fibrosis, and cirrhosis in AIH.

In the prediction of significant fibrosis, our results showed that FIB-4 had a summary sensitivity and specificity of 70% (95% CI: 54%−82%) and 70% (95% CI: 52%−83%), respectively, and a summary AUROC of 0.75 (95% CI: 0.71−0.79), while for APRI, the summary sensitivity was 90% (95% CI: 74%−97%), the summary specificity was 36% (95% CI: 21%−55%), and the summary AUROC was 0.66 (95% CI: 0.61−0.70). We found that the summary AUROC of FIB-4 for predicting significant fibrosis was higher than that of APRI. Moreover, APRI displayed a good summary sensitivity for diagnosis of significant fibrosis, but had a poor summary specificity. The summary AUROC values of FIB-4 in our study were slightly higher than that of APRI for diagnosing advanced fibrosis and cirrhosis in AIH patients (advanced fibrosis: 0.73 vs. 0.71; cirrhosis: 0.79 vs. 0.75). This suggests that FIB-4 may be a better than APRI for detecting liver fibrosis in AIH patients; however, APRI seems to be less accurate for the assessment of significant fibrosis, advanced fibrosis, and cirrhosis in AIH. In a recent multicenter prospective study by Duan et al. (19), the AUROC values of APRI and FIB-4 were 0.665 and 0.674 for diagnosing significant fibrosis, 0.670 and 0.671 for diagnosing advanced fibrosis, and 0.616 and 0.631 for diagnosing cirrhosis, in patients with chronic hepatitis B (CHB). The AUROC values of APRI and FIB-4 for the diagnosis of liver fibrosis in CHB in that study were comparable, but both were relatively lower than our present results in AIH. In the study by Wai et al. (16), the AUROC values of APRI for detecting significant fibrosis and cirrhosis in patients with chronic hepatitis C (CHC) were high (training cohort: 0.80 and 0.89; validation cohort: 0.88 and 0.94). Subsequently, two systematic reviews (22, 40) investigated the performance of APRI in predicting liver fibrosis, mainly in HCV patients. The results of previous studies demonstrated that APRI may not have great diagnostic value as initially described in HCV-infected or HCV and human immunodeficiency virus (HIV) coinfected patients. Given the prevalence of AIH, the number of original articles published on APRI and FIB-4 for evaluating different stages of liver fibrosis is limited. Thus, large scale and multicenter studies are required to further assess the comparative accuracy of APRI and FIB-4 for detecting significant fibrosis, advanced fibrosis, and cirrhosis in AIH.

Since APRI and FIB-4 were proposed as markers of liver fibrosis, they have received considerable attention for the detection of liver fibrosis induced by various causes (11). To date, several meta-analyses have investigated the diagnostic accuracy of APRI and/or FIB-4 for detecting liver fibrosis in different populations such as patients with chronic viral hepatitis and NAFLD. Of interest, in the meta-analysis by Xu et al. (21), the summary AUROC values of APRI and FIB-4 for detecting hepatitis B-related significant fibrosis were both 0.75. Similarly, in our previous study, the summary AUROC values of APRI and FIB-4 were 0.76 and 0.75, respectively, for prediction of significant fibrosis, 0.74 and 0.77, respectively, for prediction of advanced fibrosis, and 0.77 and 0.82, respectively, for prediction of cirrhosis (41). In the study by Lin et al. (22), the summary AUROC values for APRI for predicting significant fibrosis, advanced fibrosis, and cirrhosis in CHC patients were 0.77, 0.80, and 0.83, respectively. Moreover, a meta-analysis conducted by Xiao et al. (23) investigated the accuracy of these two serum indices in NAFLD patients; the reported summary AUROC values of APRI and FIB-4 were 0.76 and 0.73 for detecting significant fibrosis; 0.77 and 0.84 for detecting advanced fibrosis, respectively. In addition, APRI had a summary AUROC value of 0.76 for detecting cirrhosis. These two serum indices displayed reliable accuracy for assessing cirrhosis or advanced fibrosis in patients with chronic viral hepatitis or NAFLD.

Overall, APRI and FIB-4 did not show a high predictive accuracy for liver fibrosis in patients with AIH. In the present study, we did not assess the treatment history of the included AIH patients. Azathioprine, one of the main drugs for AIH treatment, can cause bone marrow suppression leading to thrombocytopenia (42). Both APRI and FIB-4 include platelet count. Therefore, treatment with azathioprine may have contributed to the suboptimal accuracy of these two serum indices in staging liver fibrosis in patients with AIH. Additionally, although liver biopsy is the gold standard for identifying the histological stage, potential errors in the fibrosis staging of the liver biopsies due to collapse/bridging necrosis may also have influenced the diagnostic accuracy of these tests in patients with an acute presentation of AIH.

Nevertheless, as the two most widely studied serum indices for evaluating liver fibrosis, APRI and FIB-4 offer many advantages, including wide availability, no extra costs, good reproducibility, and high applicability (43, 44). Furthermore, these two common serum indices are simple to use, and no particular expertise is required in their interpretation (45). APRI and FIB-4 tests can also be performed in an outpatient setting (45). Therefore, APRI and FIB-4 still can be considered as a choice for the diagnosis of liver fibrosis in AIH patients, especially in resource-constrained settings. For example, APRI and FIB-4 were recommended by the World Health Organization (WHO) as the preferred noninvasive tests for detection of liver fibrosis in resource-constrained settings (46). Moreover, the components of APRI and FIB-4 are routine biochemical or hematological tests that are readily available, so that these two serum indices can also be used in epidemiological research (47).

Several limitations of our meta-analysis should be acknowledged. First, only original articles published in English language were included in the meta-analysis, which may have introduced an element of bias. Second, we did not evaluate the treatment history of AIH patients. The severity of liver fibrosis may be impacted by this factor. Third, there was considerable variability among the included studies with respect to the reported cutoff values, which is likely attributable to differences in the study population as well as the variable distribution of severity. Thus, more studies are needed. Fourth, of the fourteen included studies, eight were from China, so the generalization of the present meta-analysis findings may be relatively limited. Moreover, due to the different genetic backgrounds of AIH patients with different geographic locations (48, 49), it is of great importance to investigate the performance of APRI and FIB-4 for diagnosis of liver fibrosis in patients with AIH in multicenter studies. Therefore, multicenter prospective studies are still warranted in the future. Finally, there are some intrinsic limitations in our meta-analysis, including the possibility of heterogeneity and inability to identify optimal thresholds.

In conclusion, our meta-analysis demonstrated suboptimal diagnostic performance of APRI and FIB-4 for identifying liver fibrosis in AIH patients, with mediocre sensitivity and specificity. Despite the suboptimal diagnostic accuracy of these two serum indices, their convenience of use, low cost and wide availability make them an important option for evaluation of liver fibrosis in AIH patients, especially in resource-constrained settings. Moreover, APRI and FIB-4 can also be used in epidemiological research, considering that the component parameters used are readily available. Future studies should explore novel noninvasive methods (e.g., ultrasound elastography and magnetic resonance elastography) with improved diagnostic accuracy for liver fibrosis in patients with AIH.

## Data Availability Statement

The original contributions presented in the study are included in the article/supplementary material. Further inquiries can be directed to the corresponding author.

## Author Contributions

Search of the literature and data extraction BD and CY. Drafted the manuscript BD. Performed the statistical analysis BD, CY and XY. Conceived of and supervised the study GL. All the authors edited the manuscript and provided intellectual input.

## Conflict of Interest

The authors declare that the research was conducted in the absence of any commercial or financial relationships that could be construed as a potential conflict of interest.

## Publisher’s Note

All claims expressed in this article are solely those of the authors and do not necessarily represent those of their affiliated organizations, or those of the publisher, the editors and the reviewers. Any product that may be evaluated in this article, or claim that may be made by its manufacturer, is not guaranteed or endorsed by the publisher.
